# A Case of Scleroderma With Coexisting Multiple Myeloma and Bullous Pemphigoid

**DOI:** 10.7759/cureus.66568

**Published:** 2024-08-10

**Authors:** Itır Yeğenağa, Ayli Heydari, Çağdaş Kaya, Serkan Ocakçı

**Affiliations:** 1 Nephrology, Maltepe University Faculty of Medicine, Istanbul, TUR; 2 Internal Medicine, Neurology, and Rare Diseases, Maltepe University Faculty of Medicine, Istanbul, TUR; 3 Internal Medicine, Infectious Diseases, and Rare Diseases, Maltepe University Faculty of Medicine, Istanbul, TUR; 4 Hematology, Maltepe University Faculty of Medicine, Istanbul, TUR

**Keywords:** hematology, general nephrology, bullous pemphigoid, multiple myeloma, scleroderma

## Abstract

An 83-year-old female patient presented to our nephrology outpatient clinic with complaints of weakness, edema, abdominal pain, and constipation, with a preliminary diagnosis of chronic kidney failure related to heart failure. The patient had undergone mitral valve replacement surgery 10 years prior and was diagnosed with chronic renal failure six years prior. Laboratory tests revealed mild normochromic normocytic anemia, consistently high erythrocyte sedimentation rate (ESR) above 100 mm/h, and nephrotic-range proteinuria, prompting suspicion of multiple myeloma. Further investigations, including bone marrow aspiration, confirmed the diagnosis of multiple myeloma. During follow-up, the patient began to complain of difficulty swallowing and symptoms of microstomia. Upon further questioning, it was discovered that these symptoms had been present for more than 10 years. Immunoblot tests revealed positive centromere protein B (CENP-B), suggesting a diagnosis of scleroderma. Subsequently, during follow-up, bullous lesions appeared on the patient's chest. Biopsy samples confirmed a diagnosis of bullous pemphigoid (BP). The co-occurrence of scleroderma, multiple myeloma, and superimposed BP represents a rare and noteworthy case for publication.

## Introduction

Multiple myeloma is characterized by the neoplastic proliferation of plasma cells producing monoclonal immunoglobulins. It is a clonal plasma cell cancer. The plasma cells proliferate in the bone marrow and can result in extensive skeletal destruction with osteolytic lesions, osteopenia, and pathologic fractures. However, it usually presents with unexplained anemia, hypercalcemia, some degree of kidney involvement, and a high erythrocyte sedimentation rate (ESR), especially over 100 mm/h. It constitutes slightly over 10% of all hematologic malignancies. Typically, it originates from a precancerous stage termed 'monoclonal gammopathy of undetermined significance' (MGUS), which is often asymptomatic. The MGUS progresses to myeloma or other associated malignancies at an annual rate of 1% in more than 3% of individuals over 50 [1-4].

Scleroderma, a chronic autoimmune connective tissue disease, causes damage to the skin, blood vessels, muscles, and internal organs such as the heart, lungs, and kidneys. Specific antibodies such as antinuclear antibodies, anticentromere, or anti-topoisomerase may be detected in the blood of scleroderma cases, strongly supporting autoimmune pathogenesis. Clinically, it presents with the formation of scar tissue and fibrosis in the skin and affected areas. Although the etiopathogenesis of these entities involves the activation of related inflammatory and fibrotic pathways, hereditary predisposition also plays an important role in disease development, potentially activated upon exposure to environmental stimuli and initiating inflammatory and fibrotic cascades [5]. It has been reported that an infection episode is often described before disease activation. Additionally, associations with diabetes, monoclonal gammopathy (usually IgG-κ), multiple myeloma, primary hyperparathyroidism, rheumatoid arthritis, Sjögren syndrome, and systemic lupus erythematosus have been noted.

Bullous pemphigoid (BP), characterized by autoantibodies against the dermoepidermal interface proteins BP180 and BP230, is the most prevalent autoimmune blistering disorder. Clinically, it manifests as erythematous urticarial plaques, blisters, and intense pruritus. Although numerous autoimmune disorders, such as multiple myeloma, are listed as comorbidities with BP in the literature, very few instances are associated with scleroderma, and none with both [6,7]. There have been reports linking BP to connective tissue diseases such as systemic sclerosis and scleroderma, and very rarely, with monoclonal gammopathy and multiple myeloma [8-11].

## Case presentation

An 83-year-old female was admitted to our nephrology outpatient clinic with symptoms of weakness, edema, abdominal pain, and constipation. Her medical history included a diagnosis of heart failure 10 years earlier, followed by major coronary artery bypass grafting (CABG), mitral valve replacement, annuloplasty surgery, cholecystectomy, and hysterectomy all in the same year. She was hospitalized for hemodiafiltration due to hypervolemia resulting from chronic kidney failure and heart failure. Her laboratory evaluation is presented in Tables 1-2. Elevated ESR and albumin/creatinine ratio in spot urine tests (608.27 mg/g) and protein/creatinine ratio (1556 mg/g) raised the suspicion of multiple myeloma.

**Table 1:** Laboratory results MCV: Mean corpuscular volume, CRP: C-reactive protein, ESR: Erythrocyte sedimentation rate, BUN: Blood urea nitrogen, LDH:  Lactic acid dehydrogenase, IgG: Immunoglobulin G, IgM: Immunoglobulin M, IgA: Immunoglobulin A, CA-125: Cancer antigen 125

Test	Day 1	Day 20	Day 30	Reference range
Hematocrit %	34.65	30.23	39.68	37.7-53.7
Hemoglobin g/dL	9.886	8.85	10.91	10.8-14.2
MCV fL	93.88	94.37	98.87	81.1-96
Leukocytes 10^3^/mm^3^	5.061	4.951	5.346	3.7-10.1
Thrombocytes 10^3^/uL	257.8	269.1	190.3	155-366
CRP mg/dL	0.82	1.27	0.31	0-0.5
ESR mm/h	119	101	82	0-30
Fasting glucose mg/dL	94	107	106	60-120
BUN mg/dL	43.2	52	29.9	9.8-20.1
Creatinine mg/dL	1.62	2.27	3.1	0.57-1.11
Calcium mg/dL	8.6	9.4	8.7	8.4-10.2
Sodium mmol/L	129	122	132	136-145
Potassium mmol/L	5.10	4.7	5.2	3.5-5.1
Chloride mmol/L	95.5	93.8		98.107
Phosphorus, inorganic mg/dL		7.61	3.61	2.5-4.5
LDH U/L	231		291	132-248
Albumin g/dL	3.13	2.84	3	3.2-4.6
Total protein g/dL			6.3	6-7.8
IgG mg/dL			1959	639-1349
IgM mg/dL			118	33-293
IgA mg/dL			89	69-517
Ferritin ng/mL	111.4			10-204
CA-125 U/mL	236			0-35

**Table 2:** Urine laboratory results

Test	Result	Reference range
Albumin/creatinine (spot urine) mg	608.27	<17
Total protein/creatinine mg/g	1556	0-200

**Figure 1:**
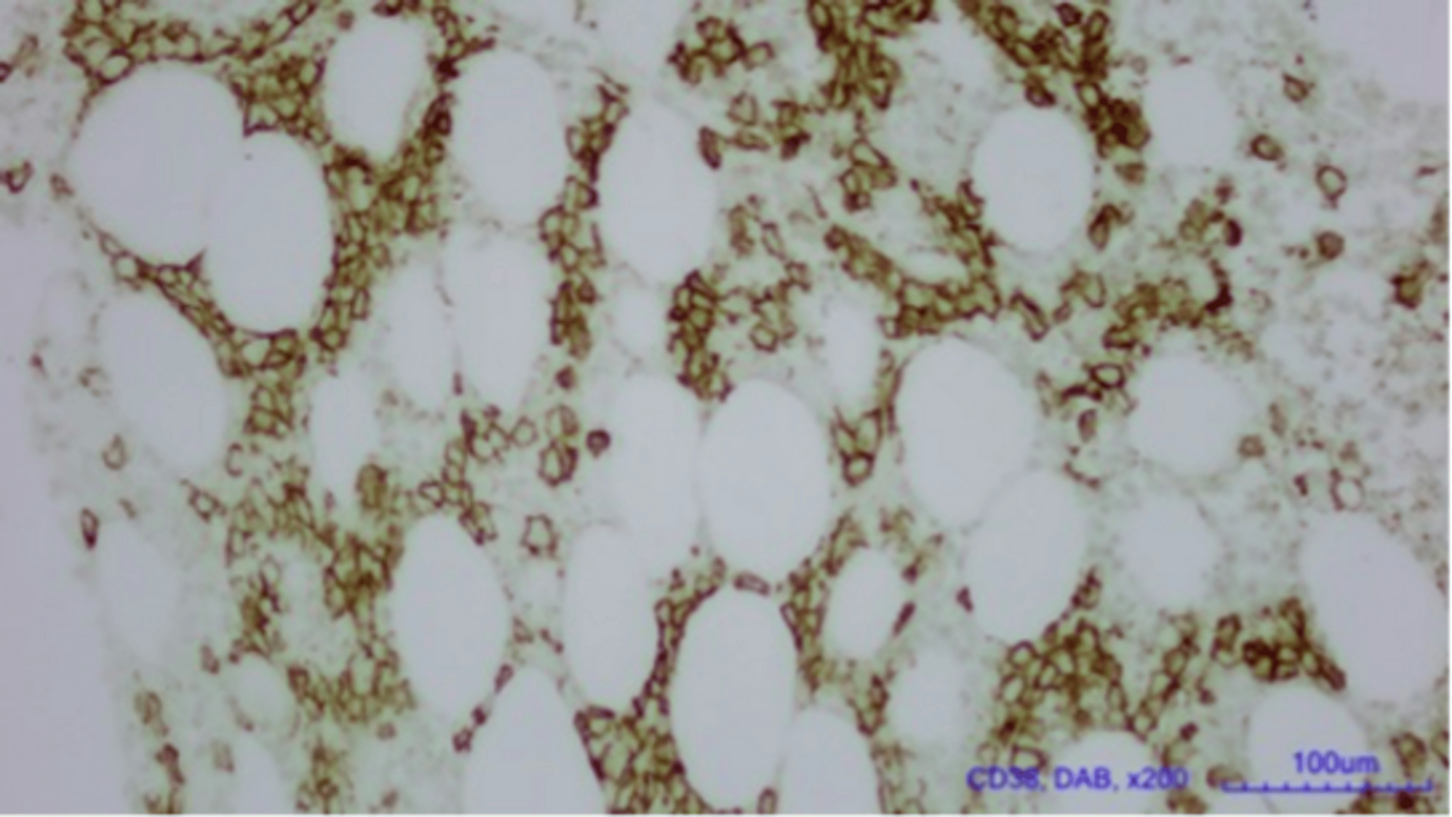
Lambda light chain seen on bone marrow biopsy (CD38, DAB, x200, 100um) Diaminobenzidine (DAB) tetrahydrochloride immunohistology staining.

**Figure 2:**
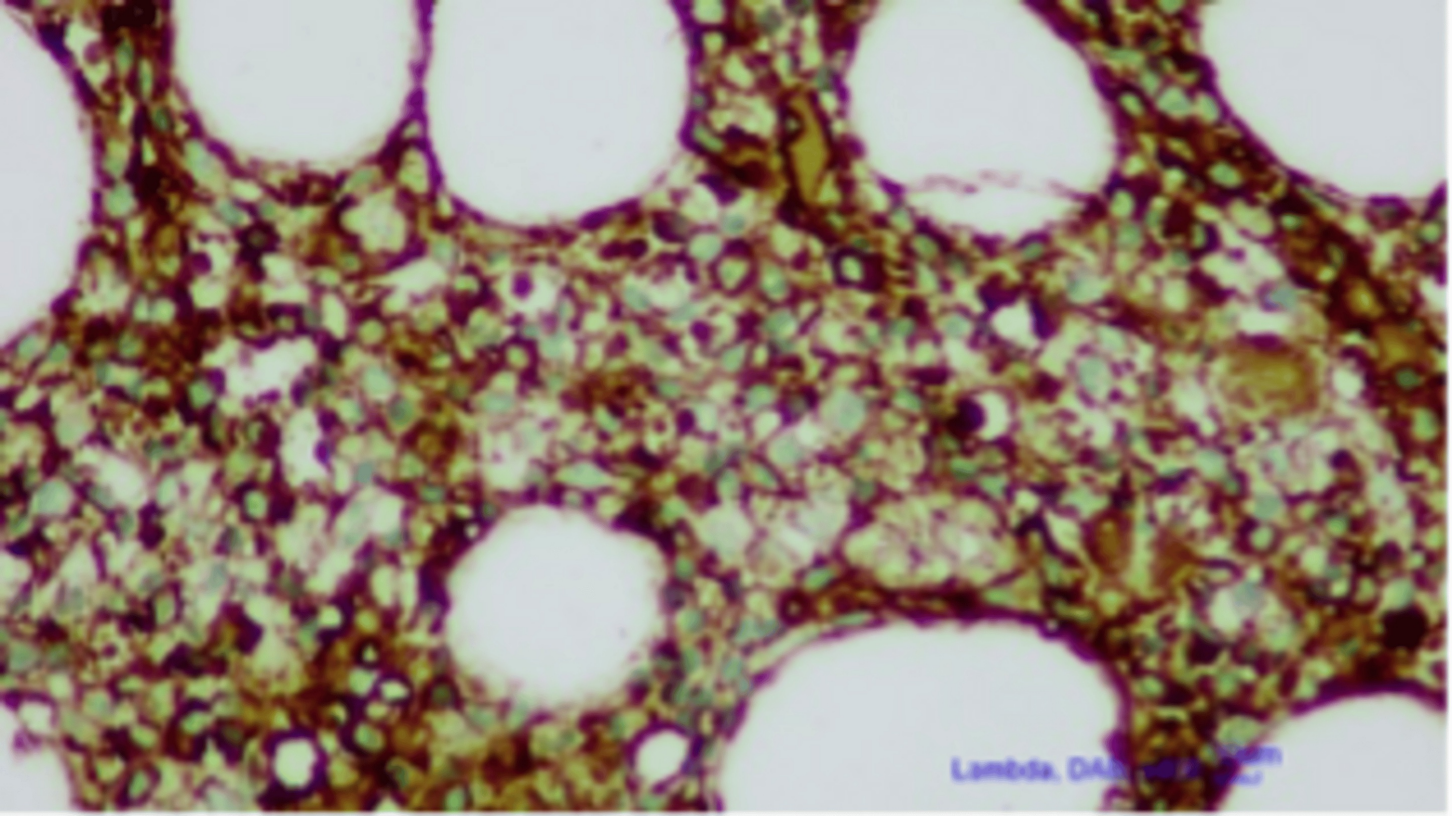
CD38 showing increased plasmacytes on bone marrow biopsy (lambda, DAB, x200, 10um) Diaminobenzidine (DAB) tetrahydrochloride hydrate immunohistology staining.

**Figure 3:**
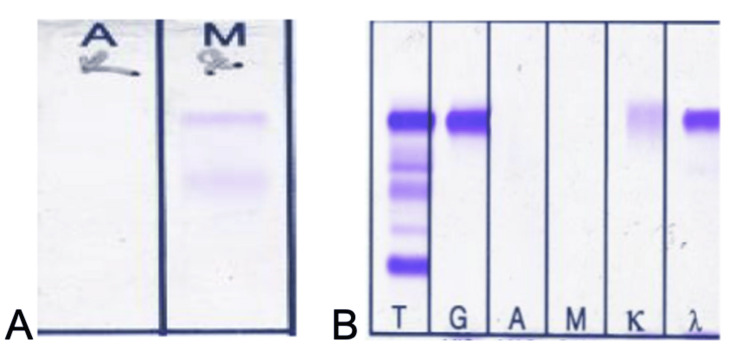
Urine immunofixation is shown in frame A and serum immunofixation in frame B

A subsequent bone marrow aspiration test revealed normocellularity with 24% plasma cells. Bone marrow biopsy, Bence Jones test, serum, and urine immunofixation tests confirmed the diagnosis of multiple myeloma (Figures 1-3).

The patient was treated with the Velcade (bortezomib), Revlimid (lenalidomide), and dexamethasone (VRd) protocol for multiple myeloma. After two cycles of chemotherapy, she began complaining of worsening difficulty swallowing and microstomia, symptoms that had been present for almost 10 years. An extractable nuclear antigen (ENA) profile test revealed a positive centromere protein B (CENP-B) immunoblot result, leading to a conclusive diagnosis of scleroderma (Tables 3-4).

**Table 3:** Bence Jones test results

Test	Results	Unit	Reference
Kappa light chain	60.30	mg/L	<15.0
Lambda light chain	310.0	mg/L	<15.0

**Table 4:** Extractable nuclear antigen profile

Test name	Result	Reference range
Anti-RNP	Negative	Negative
Anti-Sm	Negative	Negative
Anti-SS-A/Ro 60	Negative	Negative
Anti-SS-A/Ro 52	Negative	Negative
Anti-SS-B	Negative	Negative
Anti-Scl-70	Negative	Negative
PM-Scl	Negative	Negative
Anti-Jo-1	Negative	Negative
**CENP-B**	Positive	Negative
PCNA	Negative	Negative
dsDNA	Negative	Negative
Nucleosomes	Negative	Negative
Histones	Negative	Negative
PO	Negative	Negative
AMA-M2	Negative	Negative
Mi-2	Negative	Negative
KU	Negative	Negative
Anti-RNP	Negative	Negative

**Figure 4:**
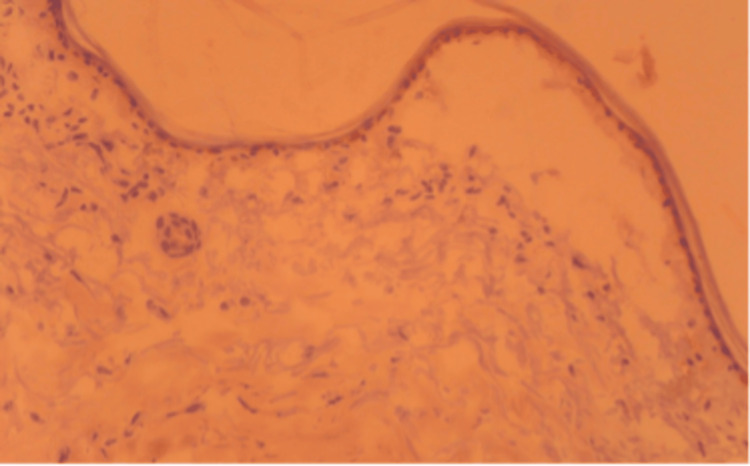
Dermoepithelial conjunction disruption of the patient with punch biopsy showing bullous lesion formation (H&E, 200x) H&E: Hematoxylin and eosin

Later, during follow-up, bullous lesions were noticed on the patient's chest. Biopsy samples from the lesion showed a thin epidermis separated from the dermoepidermal junction. Inflammation was scarce, and linear accumulations of IgA, IgM, and C3c were detected along the basal membrane. These histopathologic findings were interpreted as a diagnosis of BP (Figure 4). The patient became dependent on hemodialysis due to chronic renal failure and was also followed by the hematology department for multiple myeloma. Eight months later, she passed away due to urosepsis, which was resistant to treatment.

## Discussion

Even though there is no medical evidence, based on the patient's complaints of difficulty swallowing and microstomia, we assumed that she had been suffering from scleroderma for almost 10 years. The relationship between scleroderma and cancer has been observed for a long time, with recent reports indicating a cancer prevalence of 4% to 22% among scleroderma cases [1].

The increased incidence of cancer in scleroderma is thought to be a paraneoplastic syndrome, where the host develops an antitumor defense against cancer cells. These cancer cells, or mutated autoantigens, act as immunogens and cause autoimmunity in susceptible individuals. Risk factors for cancer development in scleroderma cases include the age of diagnosis, the presence of interstitial lung disease and pulmonary hypertension, and the extended use of calcium channel blockers [2].

In a study of 2,431 scleroderma cases and 15,141 control cases from Israel, hematological malignancies, particularly multiple myeloma, were found to be 3.03 times more frequent than in an age- and race-matched population [1,3]. A meta-analysis covering 6,641 patients with scleroderma from Australia, Northern Europe, Taiwan, and the United States found a pooled standardized incidence ratio (SIR) of 1.41 for cancer overall, with a male predominance. In this study lung, liver, and hematologic cancers were found to be increased. [2].

Paraproteinemia in scleroderma and other autoimmune diseases is well-documented. In one study, the prevalence of monoclonal immunoglobulin (mIg) in systemic sclerosis patients was found to be 13.5%, with a female predominance, and was also associated with cancer [9]. The age-adjusted risk for MGUS or multiple myeloma is reported as 4.21 (95% CI, 1.89-9.38) and 2.41 (95% CI, 1.08-5.36), respectively [9]. Molecular dysregulation and inflammation in autoimmune disorders, particularly scleroderma, may precede the clonal expansion of plasma cells, potentially contributing to the development of multiple myeloma.

The coexistence of scleroderma and multiple myeloma is very rare; only 13 cases had been reported by 2013 [6]. The case presented here was initially admitted to our clinic for heart failure and chronic renal failure. The patient's history of difficulty swallowing and microstomia led to a diagnosis of scleroderma. Although the patient did not have anti-topoisomerase 1 (anti-Scl-70), we identified positive CENP-B, which can be associated with scleroderma. Serum immunofixation electrophoresis findings showed an IgG and lambda band, and increased plasma cells in a bone marrow biopsy confirmed the diagnosis of multiple myeloma, a very rare combination.

Moreover, the appearance of bullous lesions on the chest raised the suspicion of BP, and the diagnosis was confirmed pathologically. There are reports in the literature of scleroderma cases developing BP [7,8,10]. Bullous pemphigoid is an autoimmune skin disease affecting keratinocyte adhesion to each other and the basement membrane. There is a report of a 64-year-old man with scleroderma and bullous lesions on the extremities similar to our case [8,10]. Several reports have also linked multiple myeloma to bullous dermatitis. However, various skin disorders, both autoimmune and non-autoimmune, may be associated with monoclonal gammopathies [12]. The first known case of IgA multiple myeloma presenting with intensely pruritic bullous lesions was reported in 1999 and interpreted as a cutaneous paraneoplastic condition [13]. However, the coexistence of scleroderma, multiple myeloma, and BP in a single case is extremely rare, and no previous publications have been found with this combination.

We could not detect anti-BP180 and anti-BP230 autoantibodies, but our diagnosis of BP was confirmed through biopsy, the gold standard for diagnosis. A large number of autoimmune diseases have been reported as BP comorbidities, including psoriasis, rheumatoid arthritis, lupus erythematosus, membranous nephropathy, pernicious anemia, primary biliary syndrome, and thyroiditis. In conclusion, the presence of these three rare conditions, i.e., scleroderma, multiple myeloma, and BP, in a single case is intriguing and worthy of publication. We discussed these three autoimmune conditions with a literature review.

## Conclusions

This case report describes the unusual coexistence of scleroderma, multiple myeloma, and BP in a single patient. The patient's worsening symptoms of dysphagia and microstomia, coupled with positive autoantibody findings, led to the diagnosis of scleroderma. Concurrently, the detection of monoclonal immunoglobulins confirmed the presence of multiple myeloma. The emergence of bullous lesions, followed by histopathological analysis, established the diagnosis of BP. The simultaneous occurrence of these conditions presents a unique clinical scenario, highlighting the necessity of a comprehensive and multidisciplinary approach for accurate diagnosis. This case also illustrates the potential paraneoplastic connection between autoimmune diseases and hematologic malignancies and the importance of monitoring for related comorbidities. Given the rarity of such a combination, this case adds valuable knowledge to the medical literature and may assist in the recognition and management of similar cases in the future.
